# Collapse-Related Bone Changes in Osteonecrotic Femoral Heads at Multidetector CT: Comparison between Femoral Heads with Limited and Advanced Collapse

**DOI:** 10.5334/jbsr.2735

**Published:** 2022-06-09

**Authors:** Charbel Mourad, Souad Acid, Nicolas Michoux, Anthony Awad, Bruno Vande Berg

**Affiliations:** 1 Hôpital Libanais Geitaoui- CHU, LB; 2 Cliniques Universitaires Saint Luc, BE

**Keywords:** osteonecrosis of the femoral head, hip, multidetector computed tomography (MDCT), epiphyseal collapse, cortical interruption, trabecular interruption, bone resorption

## Abstract

**Aim:**

To assess the frequency of bone changes in resected osteonecrotic femoral head (ONFH) specimens at multidetector computed tomography (MDCT) and compare their frequencies between ONFH with limited or advanced collapse.

**Method:**

Fourteen ONFH were imaged using MDCT (n = 14) and microcomputed tomography ([µCT]; n = 8). Preoperative staging was performed using radiographs and MRI. Coronal reformats of MDCT images of the specimens were analyzed using the grid overlay method. There were 2,933 grid boxes containing cortical bone and 10,596 containing trabecular bone. Two MSK radiologists assessed in every grid box the presence of interface-related sclerosis, cortical bone interruption, trabecular bone interruption, and trabecular bone resorption. The frequency of grid boxes with bone changes at MDCT was calculated and compared between ONFH with limited (<1.5 mm) or advanced (≥1.5 mm) collapse.

**Results:**

For both readers R1 and R2, there were 1111/10596 (10.5%) and 1362/10596 (12.9%) grid boxes with interface-related bone sclerosis, 557/2933 (19%) and 413/2933 (14.1%) with cortical bone interruption, 796/10596 (7.5%) and 665/10596 (6.3%) with trabecular bone interruption, and 331/10596 (3.1%) and 595/10596 (5.6%) with trabecular bone resorption. The frequency of grid boxes with cortical interruption and trabecular bone resorption was significantly higher in ONFH with advanced than in ONFH with limited collapse. There was no significant difference in frequency of grid boxes with trabecular interruption and interface-related bone sclerosis between ONFH with advanced or limited collapse.

**Conclusion:**

Cortical interruption and trabecular resorption, but not trabecular interruption, were more frequent in osteonecrotic femoral heads with advanced than with limited collapse.

## Introduction

The occurrence of collapse in osteonecrotic femoral heads (ONFH) is a pivotal step towards osteoarthritis and subsequent total hip replacement. The detection of collapse relies on conventional radiography and MRI [1]. More recently, multidetector computed tomography (MDCT) has been shown to be superior to MRI and radiographs in the detection of subchondral fractures [2,3,4]. There is a need to better understand bone changes associated with progressive collapse. The aim of this study was to determine the frequency and topology of bone changes in a quantitative analysis performed on MDCT images of resected ONFH specimens using the grid overlay method, and to compare their frequency between femoral heads with limited or advanced collapse.

## Material and Methods

### 1- Patients’ population

The Institutional Review Board approved the study, with the informed consent of the patients being waived because the study was performed on femoral head specimens that were resected for total hip replacement. An MSK radiologist with four years of experience collected 14 femoral head (FH) specimens with osteonecrosis (ON) from 13 patients: 8 men (46.9 years ±13.9) and 5 women (68.4 years ±16.05). These specimens were resected during hip replacement. The FH specimens were fixated in formalin. In a preliminary study on eight FH specimens, the sensitivity and specificity of *in vitro* MDCT were calculated in comparison to µCT (see online supplemental material).

### 2- Staging of ONFH and measurement of articular surface collapse

Staging of ONFH was performed on radiographs and MRI according the ARCO classification [4]. Collapse of the articular surface was measured using the best fitting concentric circle technique [5]. The median collapse was 1.5 mm [1.1–2.1 mm, 95% CI]. There were 10 stage 3-As, 3 stage 3-Bs, and 1 stage 4 ONFH.

### 3- Data acquisition and grid overlying

**Figure 1:**
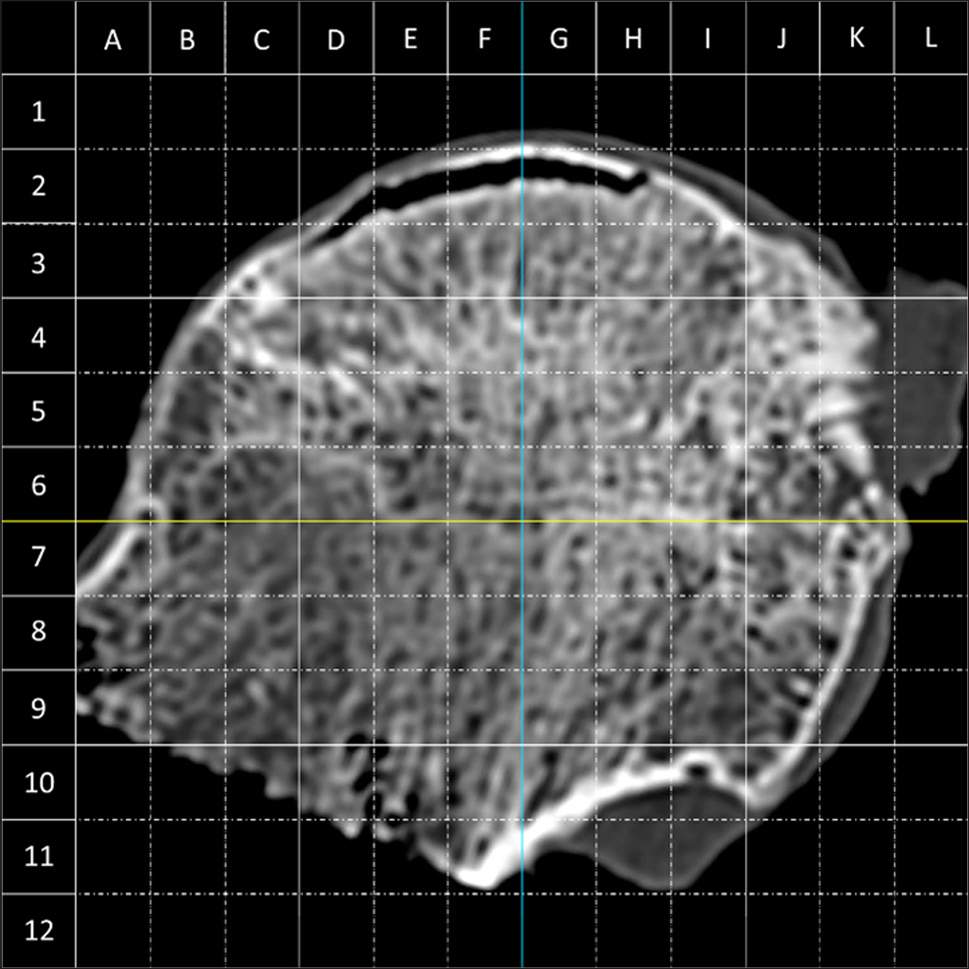
A 12 × 12 transparent grid was overlayed on coronal MDCT images. Image analysis was performed in every 4 × 4 mm grid box. As an example, grid boxes D2, D3, E2, F2, G2 and H2 contained collapse-related trabecular interruption.

Fourteen resected FH specimens were scanned on a 40-row MDCT (Siemens Somatom Definition 40, Erlangen, Germany). One radiologist with four years of experience viewed the MDCT images on an image viewer with multiplanar capacity. Coronal reformats were obtained and segmented by overlaying a transparent 12 × 12 grid. The anonymized images with the overlaid grid were uploaded on an in-house developed software that enables image analysis (Figure 1 and E1). Further details are provided in online supplemental material.

### 4- Image analysis and lesion definition

Images were independently analyzed by an MSK radiologist with 30 years of experience (R1), and an MSK radiologist with four years of experience (R2). The reading process was performed in 4 × 4 mm grid boxes using the grid overlay method on in-house developed software (see online supplemental material).

**Figure 2:**
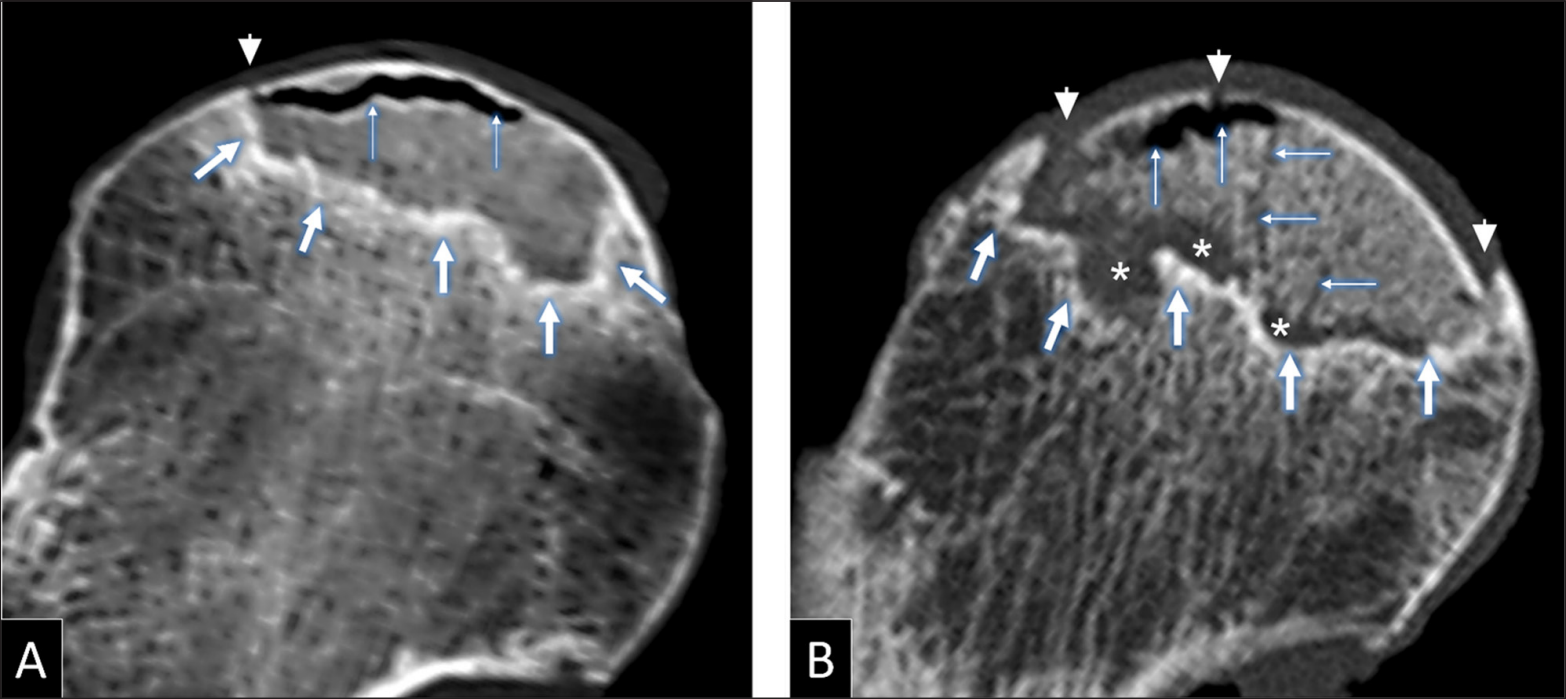
Coronal MDCT reformats of resected femoral head specimens of **(A)** a 59-year-old woman and **(B)** an 85-year-old woman, showing interface-related bone sclerosis (thick arrows) and collapse-related bone changes: cortical interruption (arrowheads), trabecular interruption (thin arrows) and bone resorption (asterisks in B).

**Figure 3:**
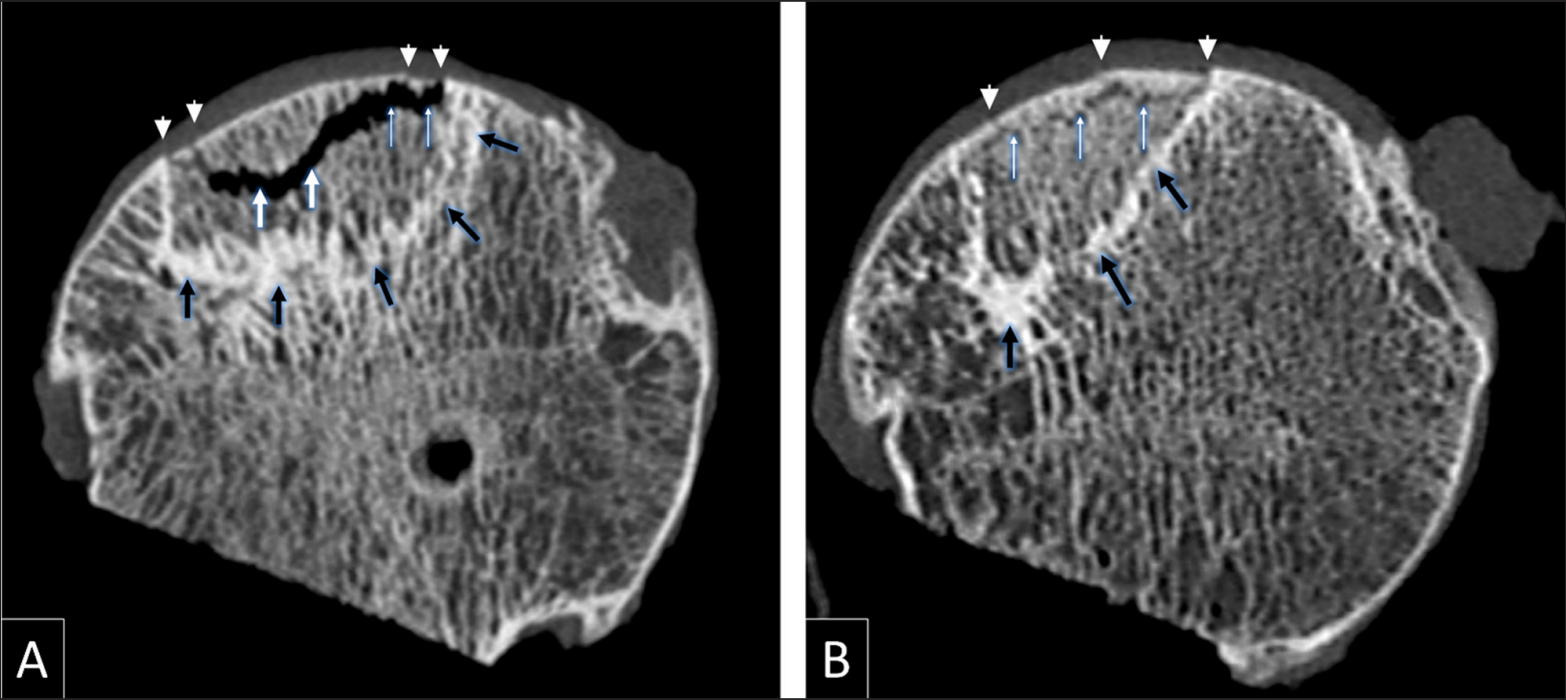
**(A)** and **(B)** Coronal MDCT reformats of a resected femoral head specimen in a 62-year-old man, showing collapse-related trabecular interruption in the superficial (thin white arrows) and deep layers (thick white arrows) of the trabecular bone. Also note cortical interruption (arrowheads) and interface-related bone sclerosis (black arrows).

Bone changes at MDCT were defined as follows: interface-related trabecular sclerosis corresponded to thickened trabeculae with a band-like distribution and a concave orientation towards the articular surface. Cortical bone interruption corresponded to a focal interruption of the subchondral bone plate with or without deformity (Figure 2). Trabecular bone interruption corresponded to an interruption of two or more contiguous trabeculae, having irregular, sharp, or angular margins with or without a gas-filled cleft or a dense linear band corresponding to trabecular crushing (Figures 2, 3). Trabecular bone resorption corresponded to a lucent zone devoid of mineralized content, with smooth or rounded margins (Figure 2).

Limited FH collapse was defined by the presence of a depression of the FH contours <1.5 mm, and advanced FH collapse by the presence of a depression ≥1.5 mm.

### 5- Statistical analysis

Data output was automatically generated on Excel sheets. Frequency of grid boxes with bone changes was compared between FH with limited and advanced collapse using Chi-squared test. Interobserver agreement was computed using Cohen’s Kappa and interpreted according to the scale proposed by Altman [6]. A *p*-value <0.05 was regarded as statistically significant. Statistical analyses were performed by using MedCalc Statistical Software version 19.2.6 (MedCalc Software bv, Ostend, Belgium; https://www.medcalc.org; 2020).

## Results

### 1- Frequency of grid boxes with bone changes at MDCT

There were 2,933 grid boxes containing cortical bone and 10596 grid boxes containing trabecular bone on the MDCT reformats. For R1, there were 557/2933 (19%) with cortical bone interruption, 796/10596 (7.5%) with trabecular bone interruption, and 331/10596 (3.1%) with trabecular bone resorption. Results for R2 are given in Table 1.

**Table 1:** Frequency of grid boxes with cortical and trabecular bone changes on coronal MDCT reformats of 14 femoral head specimens.


		R1	R2

**Necrosis-relatedbone changes**	**Necrotic lesion**	3442/10596 (32.5%)	3666/10596 (34.6%)

	**Trabecular sclerosis**	1111/10596 (10.5%)	1362/10596 (12.9%)

**Collapse-relatedbone changes**	**Cortical interruption**	557/2933 (19%)	413/2933 (14.1%)

	**Trabecular interruption**	796/10596 (7.5%)	665/10596 (6.3%)

	**Trabecular resorption**	331/10596 (3.1%)	595/10596 (5.6%)


R1 and R2 represent two readers.

### 2- Topology of grid boxes with necrotic lesion and collapse-related bone changes at MDCT

For R1, grid boxes with collapse-related bone changes (CRBC) at MDCT were more frequent in the anterior, central, and superior thirds of the FH (Table 2). Grid boxes with trabecular interruption were more frequent in the superficial (448/2621; 17.1%) than in the deep layer (348/7975; 4.4%). Grid boxes with trabecular resorption were more frequent in the deep (281/7975; 3.5%) than in the superficial layer (50/2621; 1.9%). Detailed results for R1 and R2 are provided in Table 2.

**Table 2:** Frequency of grid boxes with CRBC and with necrotic lesion in different thirds regions of the FH in the coronal plane on MDCT reformats.


		ANTERO-POSTERIOR DIRECTION				MEDIO-LATERAL DIRECTION				CRANIO-CAUDAL DIRECTION				RADIAL DIRECTION		
			
		ANT	MID	POST	P	MED	CENT	LAT	P	SUP	MID	INF	P	SUPERFICIAL	DEEP	P

**R1**	NecroticLesion*	782/2159 (36.2%)	1345/4354 (30.9%)	623/2419 (25.8%)	P^1^ < 0.001 P^2^ < 0.001 P^3^ < 0.001	681/2427 (28.1%)	1582/4345 (36.4%)	487/2160 (22.5%)	P^4^ < 0.001 P^5^ < 0.001 P^6^ < 0.001	1713/2273 (75.4%)	1030/4632 (22.2%)	7/2027 (0.3%)	P^7^ < 0.001 P^8^ < 0.001 P^9^ < 0.001	823/2232 (36.9%)	1927/6700 (28.8%)	P < 0.001

	**Trabecularsclerosis**	324/2562 (12.6%)	509/5134 (9.9%)	278/2900 (9.6%)	P^1^ < 0.001 P^2^ < 0.001 P^3^ = 0.664	273/2867 (9.5%)	587/5125 (11.45%)	251/2604 (9.6%)	P^4^ = 0.007 P^5^ = 0.090 P^6^ = 0.013	422/2671 (15.8%)	650/5447 (11.9%)	39/2478 (1.6%)	P^7^ < 0.001 P^8^ < 0.001 P^9^ < 0.001	203/2621 (7.7%)	908/7975 (11.4%)	P < 0.001

	**Corticalinterruption**	213/830 (25.7%)	236/1199 (19.7%)	108/904 (11.9%)	P^1^ = 0.001 P^2^ < 0.001 P^3^ < 0.001	131/1357 (6.7%)	252/846 (29.8%)	174/730 (23.8%)	P^4^ < 0.001 P^5^ < 0.001 P^6^ = 0.007	486/1388 (35%)	71/941 (7.5%)	0/604 (0%)	P^7^ < 0.001 P^8^ < 0.001 P^9^ < 0.001	NA	NA	

	**Trabecularinterruption**	243/2562 (9.5%)	404/5134 (7.9%)	149/2900 (5.1%)	P^1^ = 0.02 P^2^ < 0.001 P^3^ < 0.001	160/2867 (5.6%)	448/5125 (8.7%)	188/2604 (7.2%)	P^4^ < 0.001 P^5^ = 0.016 P^6^ = 0.023	693/2671 (25.9%)	99/5447 (1.8%)	4/2478 (0.2%)	P^7^ < 0.001 P^8^ < 0.001 P^9^ < 0.001	448/2621 (17.1%)	348/7975 (4.4%)	P < 0.001

	**Trabecularresorption**	162/2562 (6.3%)	125/5134 (2.4%)	44/2900 (1.5%)	P^1^ < 0.001 P^2^ < 0.001 P^3^ = 0.007	59/2867 (2.1%)	204/5125 (4%)	68/2604 (2.6%)	P^4^ < 0.001 P^5^ = 0.221 P^6^ = 0.002	171/2671 (6.4%)	143/5447 (2.6%)	17/2478 (0.7%)	P^7^ < 0.001 P^8^ < 0.001 P^9^ < 0.001	50/2621 (1.9%)	281/7975 (3.5%)	P < 0.001

**R2**	NecroticLesion*	884/2159 (40.9%)	1323/4354 (30.4%)	607/2419 (25.1%)	P^1^ < 0.001 P^2^ < 0.001 P^3^ < 0.001	685/2427 (28.2%)	1585/4345 (36.5%)	544/2160 (25.2%)	P^4^ < 0.001 P^5^ = 0.022 P^6^ < 0.001	1766/2273 (77.7%)	1036/4632 (22.4%)	12/2027 (0.6%)	P^7^ < 0.001 P^8^ < 0.001 P^9^ < 0.001	897/2232 (40.2%)	1917/6700 (28.6%)	P < 0.001

	**Trabecularsclerosis**	421/2562 (16.4%)	616/5134 (12%)	325/2900 (11.2%)	P^1^ < 0.001 P^2^ < 0.001 P^3^ = 0.284	332/2867 (11.6%)	662/5125 (12.9%)	368/2604 (14.1%)	P^4^ = 0.091 P^5^ = 0.006 P^6^ = 0.014	590/2671 (22.1%)	732/5447 (13.4%)	40/2478 (1.6%)	P^x^ < 0.001 P^y^ < 0.001 P^z^ < 0.001	317/2621 (12.1%)	1045/7975 (13.1%)	P = 0.185

	**Corticalinterruption**	168/830 (20.2%)	179/1199 (14.9%)	66/904 (7.3%)	P^1^ = 0.002 P^2^ < 0.001 P^3^ < 0.001	112/1357 (8.3%)	186/846 (22%)	115/730 (15.75%)	P^4^ < 0.001 P^5^ < 0.001 P^6^ = 0.002	350/1388 (25.2%)	60/941 (6.4%)	3/604 (0.5%)	P^7^ < 0.001 P^8^ < 0.001 P^9^ < 0.001	NA	NA	

	**Trabecularinterruption**	228/2562 (8.9%)	306/5134 (6%)	131/2900 (4.5%)	P^1^ < 0.001 P^2^ < 0.001 P^3^ = 0.004	108/2867 (3.8%)	432/5125 (8.4%)	125/2604 (4.8%)	P^4^ < 0.001 P^5^ = 0.068 P^6^ < 0.001	582/2671 (21.8%)	83/5447 (1.5%)	0/2478 (0%)	P^7^ < 0.001 P^8^ < 0.001 P^9^ < 0.001	389/2621 (14.8%)	276/7975 (3.5%)	P < 0.001

	**Trabecularresorption**	243/2562 (9.5%)	248/5134 (4.8%)	104/2900 (3.6%)	P^1^ < 0.001 P^2^ < 0.001 P^3^ = 0.011	116/2867 (4.1%)	334/5125 (6.5%)	145/2604 (5.6%)	P^4^ < 0.001 P^5^ = 0.01 P^6^ = 0.12	353/2671 (13.2%)	238/5447 (4.4%)	4/2478 (0.2%)	P^7^ < 0.001 P^8^ < 0.001 P^9^ < 0.001	110/2621 (4.2%)	485/7975 (6.1%)	P < 0.001


*Frequency of grid boxes containing the necrotic lesion was calculated in 12 instead of 14 ONFH because the interface was only partially visible. The denominators represent the total number of grid boxes in every region. (p^1^) Anterior versus Middle; (p^2^) Anterior versus Posterior; (p^3^) Middle versus posterior. (p^4^) = Medial versus Central; (p^5^) = Medial versus Lateral; (p^6^) = Central versus Lateral. (p^7^) = Superior versus Middle; (p^8^) = Superior versus Inferior; (p^9^) = Middle versus inferior.

For R1, grid boxes with cortical and trabecular bone changes were more frequent within than outside the necrotic lesion (p < 0.001). Detailed results for R1 and R2 are provided in Table 3.

**Table 3:** Comparison of frequency of grid boxes with CRBC on coronal MDCT reformats within or outside the osteonecrotic lesions*.


	COLLAPSE-RELATED BONE CHANGES	OUTSIDE NECROTIC LESION	WITHIN NECROTIC LESION	P VALUE

**R1**	**Cortical bone interruption**	42/1507 (2.8%)	415/979 (42.4%)	p < 0.001

	**Trabecular bone interruption**	18/6182 (0.3%)	648/2750 (23.6%)	p < 0.001

	**Trabecular bone resorption**	20/6182 (0.3%)	246/2750 (8.9%)	p < 0.001

**R2**	**Cortical bone interruption**	24/1507 (1.6%)	315/979 (32.2%)	p < 0.001

	**Trabecular bone interruption**	3/6118 (0.05%)	544/2814 (19.3%)	p < 0.001

	**Trabecular bone resorption**	3/6118 (0.05%)	519/2814 (18.4%)	p < 0.001


*Frequency of grid boxes containing the necrotic lesion was calculated in 12 instead of 14 ONFH because the interface was only partially visible.

### 3- Comparison of frequency of grid boxes with necrotic lesions and CRBC at MDCT between limited and advance collapse

For R1, grid boxes with cortical interruption were more frequent in FH with collapse ≥1.5 mm (20.49%) than in FH with collapse <1.5 mm (17.02%) (p = 0.018). There was no statistical difference in the frequency of grid boxes with trabecular interruption between FH with collapse ≥1.5 mm (7.13%) than <1.5 mm (8%) (p = 0.094). Grid boxes with trabecular resorption were more frequent in FH with collapse ≥1.5 mm (3.88%) than <1.5 mm (2.15%) (p < 0.001). Detailed results for R1 and R2 are provided in Table 4.

**Table 4:** Comparison of frequency of grid boxes with necrotic lesions and CRBC at MDCT between ONFH with limited and advanced collapse.


FREQUENCY OF GRID BOXES CONTAINING	R1			R2		
	
	**COLLAPSE** **<1.5 mm**	**COLLAPSE** **≥1.5 mm**	**P VALUE**	**COLLAPSE** **<1.5 mm**	**COLLAPSE** **≥1.5 mm**	**P VALUE**

**Necrotic lesion**	1481/4610(32.13%)	1961/5986(32.76%)	P = 0.503	1460/4610(31.67%)	2206/5986(36.85%)	p < 0.001

**Interface-relatedTrabecular sclerosis**	499/4610 (10.82%)	612/5986(10.22%)	P = 0.318	577/4610(12.51%)	785/5986(13.11%)	P = 0.360

**Cortical bone interruption**	216/1269(17.02%)	341/1664(20.49%)	P = 0.018	121/1269(9.54%)	292/1664(17.55%)	p < 0.001

**Trabecular bone interruption**	369/4610(8%)	427/5986 (7.13%)	P = 0.094	299/4610(6.49%)	366/5986(6.11%)	P = 0.443

**Trabecular bone resorption**	99/4610(2.15%)	232/5986(3.88%)	p < 0.001	214/4610(4.64%)	381/5986(6.36%)	p < 0.001


## Discussion

In the current study, we assessed bone changes in ONFH specimens using MDCT and found that grid boxes with necrotic lesion and CRBC predominated in the anterior, central, and superior parts of ONFH specimens. We also found that grid boxes with cortical interruption and trabecular bone resorption were more frequent in femoral heads with advanced collapse than in those with limited collapse, with no difference in frequency of bone interruption according to the degree of collapse. These observations suggest that progression of collapse could be more associated with the development of cortical bone fracture and bone resorption than with trabecular bone interruption.

First, the observation that trabecular bone interruption predominated in the superficial layer of the necrotic lesion could be explained by the concentration of mechanical stress on the surface or by its association with cortical interruption [7,8,9,10,11]. The predominance of trabecular bone resorption in the deep regions of the necrotic lesions is associated with the repair process that include increased bone remodeling in vascularized regions surrounding and invading the lesion [11,12].

Second, the fact that cortical bone interruption was more frequent in ONFH with advanced than with limited collapse is associated with marked deformity of the subchondral bone plate that results from collapse. The observation that bone resorption was more frequent with more advanced collapse suggests that resorption could develop during progressive failure of the FH and is not associated with early fracture [9,13].

Third, the lack of difference in frequency of trabecular interruption according to the degree of collapse could contradict the general idea that links trabecular fracture with collapse. Trabecular fracture formation could differ from collapse, similar to what is observed in rocks [14]. In ONFH, stress distribution is altered by progressive collapse and development of trabecular fracture can be dissociated from that of cortical fracture [15]. The hypothesis that progression of collapse is associated with bone resorption and cortical fracture but not with trabecular bone fracture deserves further analysis.

Our study has many limitations. In addition to a limited number of specimens, histological confirmation of the findings was not performed. Since µCT can accurately detect cortical and trabecular microfractures in comparison with histology [16,17], we obtained µCT examinations in eight specimens and demonstrated that MDCT had a high specificity to detect cortical fractures, trabecular fracture, resorption, and sclerosis in comparison with µCT (see supplemental material).

In conclusion, cortical and trabecular bone interruption and trabecular bone resorption are found in collapsed ONFH and their distribution parallels that of the necrotic lesions. Grid boxes with cortical bone interruption and trabecular bone resorption but not with trabecular bone interruption are more frequent in ONFH with advanced than with limited collapse.

## Figures and Tables

**Table:** 

ARCO:	Association Research Circulation Osseous
CRBC:	collapse-related bone changes
FH:	femoral head
MDCT:	multidetector computed tomography
ON:	osteonecrosis
ONFH:	osteonecrotic femoral heads
µCT:	Microcomputed tomography
